# Special Issue “Lightweight Structural Materials for Automotive and Aerospace”

**DOI:** 10.3390/ma15207089

**Published:** 2022-10-12

**Authors:** Frank Czerwinski

**Affiliations:** CanmetMATERIALS, Natural Resources Canada, Hamilton, ON L8P 0A5, Canada; frank.czerwinski@nrcan-rncan.gc.ca

## Foreword

Modern transport represents a vital part of the global economy, but it is also a significant source of pollutants, contributing 13% of overall greenhouse gas and 25% of CO_2_ emissions coming from the combustion of fossil fuels. An application of materials with high strength-to-weight ratios in transportation vehicles, commonly known as lightweighting, is an important strategy for improving the fuel economy and reducing harmful pollution. Although the lighter vehicle is always more efficient, irrespective of its propulsion, the emergence of electric vehicles and needs to offset the weight of batteries and electric motors exert even more pressure on lightweighting. 

At present, lightweighting is becoming the major trend, reaching many industrial sectors associated not only with all forms of transportation but more broadly with civil infrastructure, manufacturing, and clean energy technologies. In fact, the lightweighting objectives are not exclusively focused on the reduction of weight but also cover other aspects involving the structural efficiency, as well as the economic and environmental impact. Although lightweighting is not a new concept and aerospace has been on the lightweight path since its origin, while other sectors have also pursued it for decades, it is re-emerging as the mature, enormous growth course that is driven by sustainability, cost, and performance.

Lightweight materials are the essence of the weight reduction strategy and their typical list includes aluminum, magnesium, beryllium, titanium, titanium aluminides, structural ceramics, and composites with polymer, metal, and ceramic matrices. Many of them, along with the essential alloying additions, are classified as the critical minerals, which have a significant economic or national importance and a supply chain vulnerable to disruption. 

This Special Issue “Lightweight Structural Materials for Automotive and Aerospace” covers selected aspects of current lightweighting trends in transportation, emphasizing both the development of modern alloys and examples of their industrial implementations.

